# A Comprehensive Analysis and Review of Artificial Intelligence in Anaesthesia

**DOI:** 10.7759/cureus.45038

**Published:** 2023-09-11

**Authors:** Meghna Singhal, Lalit Gupta, Kshitiz Hirani

**Affiliations:** 1 Department of Anesthesiology and Critical Care, Maulana Azad Medical College, Delhi, IND; 2 Department of Anesthesiology and Critical Care, University College of Medical Sciences and Guru Teg Bahadur Hospital, Delhi, IND

**Keywords:** artificial intelligence and robotics in healthcare, ai and machine learning, machine learning, legal and ethical issues, technical limitations, record-keeping automation, individualisation of drug dosage, prediction of adverse events, patient monitoring, anaesthesia

## Abstract

In the field of anaesthesia, artificial intelligence (AI) has become a game-changing technology. Applications of AI include keeping records, monitoring patients, calculating and administering drugs, and carrying out mechanical procedures. This article explores the current uses, challenges, and prospective applications of AI in anaesthesia practices. This review discusses AI-supported systems like anaesthesia information management systems (AIMS), mechanical robots for carrying out procedures, and pharmacological models for drug delivery. AIMS has helped in automated record-keeping, predicting bad events, and monitoring the vital signs of the patient. Their application has a vital role in improving the efficacy of anaesthesia management and patient safety.

The application of AI in anaesthesia comes with its own unique difficulties. Noteworthy obstacles include issues with data quantity and quality, technical limitations, and moral and legal dilemmas. The key to overcoming these barriers is to set guidelines for the ethical use of AI in healthcare, improve the reliability and comprehension of AI systems, and certify the health data precision and security.

AI has very bright potential. Exciting future directions include developments in AI and machine learning thus development of new applications, and the possible enhancement in training and education. Potential research areas include the application of AI to chronic disease management, pain management, and the reinforcement of anaesthesiologists' education. AI could be used to design authentic lifelike training simulations and individualized student feedback systems, hence transforming anaesthesia education and training methodology. For this review, we conducted a PubMed, Google Scholar, and Cochrane Database search in 2022-2023 and retrieved articles on AI and its uses in anaesthesia.

Recommendations for future research and development include strengthening the safety and reliability of health data, building a better understanding of AI systems, and looking into new areas of use. The power of AI can be used to innovate anaesthesia practices by concentrating on these areas.

## Introduction and background

Artificial intelligence (AI) as a multidisciplinary computerized research field not only focuses on the expansion and generation of intelligent computer algorithms to carry out simple to complex tasks that traditionally require human intelligence. These tasks ranged from the intellectual ability of learning and critical thinking to problem-solving, perceiving, and development of a comprehending philological aspect of language [1,2]. AI can be best defined as a cluster of highly sophisticated technologies used in many different fields. It comprehends a number of subfields, including robotics, natural language processing, and machine learning, which allow computers to evolve by understanding and interpreting human language, as well as integrating new tasks for which they were not initially programmed [3].

AI has been a transformational force in the field of anaesthesia. Many surgical interventions need the administration of anaesthesia, which calls for a high level of skill and accuracy. An anaesthesiologist's responsibilities include evaluating patients prior to surgery, assuring their safety throughout the procedure, and guaranteeing their comfort following it. AI can help anaesthesiologists with these activities, thus improving patient care, lowering expenses, and boosting productivity. AI can assist in automating record-keeping, predicting and preventing adverse occurrences, individualizing prescription dosages, and monitoring patient vital signs [4-6]. By doing this, anaesthesiologists can concentrate more on patient care rather than mundane activities [7]. As a result, AI is crucial to anaesthetics, and it has a wide range of possible uses in the future.

For this review, we conducted a PubMed, Google Scholar, and Cochrane Database search and retrieved articles using keywords such as AI and its application in anaesthesia, pharmacological, cognitive, and mechanical robot’s in anaesthesia, simulation in anaesthesia, and pain management using AI, anaesthesia information management system (AIMS), and smart anaesthesia manager (SAM).

## Review

History of AI in anaesthesia

The history of AI in anaesthesia is an interesting one spanning many decades, and it is constantly evolving with time. Early researchers concentrated on developing expert algorithms, which are computer programs made to resemble the judgment of a human expert. These systems were designed for the purpose of anaesthesia to aid anaesthesiologists in drug administration and patient monitoring. For instance, the "Diprifusor" system, a pioneering example of AI in anaesthesia, was created in the late 90s and is a propofol target-controlled infusion system [8,9]. This system uses the pharmacokinetic properties of the propofol for the drug's infusion, sustaining the desired plasma concentration of the drug. Another early AI system, the "closed loop anaesthesia drug administration" (CLAD) system, used a fuzzy logic controller to deliver propofol and remifentanil while maintaining the required depth of anaesthesia [10,11].

As technology advanced, attention turned toward more complex AI programs that employ machine learning and deep learning. These processes gave AI systems the ability to learn from data, develop over time, and become more reliable and efficient [12]. These developments lead to the construction of AI systems that are capable of performing difficult tasks like anticipating patient reactions to drugs used in anaesthesia practices, recognizing risk factors associated with complications, and even helping to manage anaesthesia during surgery [12-15]. For instance, the AIMS employs AI to collect, store, and analyze patient data, giving anaesthesiologists real-time information to help in better patient management [16]. Another illustration is SAM, an AI system that works in conjunction with AIMS to analyze patient data and recommends suggestions for anaesthetic management using machine learning technology [17]. These contemporary AI technologies have enhanced safety and effectiveness and have expanded opportunities for anaesthetic research and innovation.

AI's present applications in anaesthesia

Numerous applications of AI in the field of anaesthesia have considerably improved patient safety and treatment quality. The monitoring of patients' vital signs is one of the most important uses of AI in anaesthesia [6]. A patient's heart rate, blood pressure, oxygen saturation, and other vital signs can be continuously monitored by AI systems, which can also notify the anaesthesiologist of any changes that might be problematic [17]. For example, the Philips "IntelliVue" patient monitor employs AI algorithms to enable real-time monitoring of vital signs, allowing anaesthesiologists to quickly identify and react to changes in the patient's condition [18]. AI technologies can help anaesthesiologists make educated decisions and take the necessary action by delivering real-time, accurate information about the patient's state, improving patient safety [17,19].

Predicting and preventing unfavorable outcomes is an important use of AI in anaesthesia. Serious effects may result from adverse anaesthetic events such as hemodynamic instability or breathing problems [2,20]. Data from past treatments can be analyzed by AI systems to spot patterns and trends that can point to a higher risk of problems [21].

Cognitive Robots

For instance, AIMS makes use of AI algorithms to analyze patient data and foresee the likelihood of unfavorable occurrences [2,20]. These devices enable anaesthesiologists to take preventive action, such as modifying the anaesthesia plan or increasing the frequency of monitoring, by identifying high-risk patients. A variety of AIMS systems are available for many uses like preoperative evaluation, drug calculations, intraoperative monitoring, and clinical decision support systems [22-25]. AIMS helps in record keeping, collection and storage, quality assurance, and decreasing billing disparities [4,26-27]. Examples of commercially available AIMS systems by different vendors are Innovian Anaesthesia in Drager, Centricity Anaesthesia in GE Healthcare, and Compu Record in Philips Medical Systems (Table 1) [16].

**Table 1:** Cognitive robots in anaesthesia AIMS: anaesthesia information management system, SAM: smart anaesthesia manager

Systems	Uses
AIMS	Can be helpful in preoperative evaluations [22]
	Can assist in intraoperative monitoring and highlighting key changes [23,24]
	Helps clinicians by providing a clinical decision support system [25]
	Helps in research by improving record-keeping, data collection, and storage [4]
	Quality assurance [26]
	Accurate billing of the patients [27]
SAM	Improved compliance to drugs like intraoperative antibiotics and perioperative beta blocker therapy
	Decreased inadvertent gaps in patient vital monitoring
	Improved billing of the procedure done

SAM is a system developed to work with AIMS systems to improve clinical decisions and decrease billing disparities. SAM uses the AIMS database to provide real-time information to support clinical decisions through pop-up messages on AIMS monitors. It has improved compliance with drugs, improved timely patient monitoring, and ensured proper billing [17]. It has also been found that they improved compliance with the antibiotic initial dose and re-dose, beta blocker protocol compliance, and reduction in inadvertent gaps (>15 min) in blood pressure monitoring (Table 1).

Pharmacological Models

AI is also essential for customizing anaesthetic medication doses. Because every patient is different, the amount of anaesthesia required can also vary greatly depending on a patient's age, weight, BMI, medical history, and present state of health. These variables can be analyzed by AI systems to identify the best dosage for each patient. AI can ensure that each patient receives the appropriate dose of anaesthesia by personalizing medicine dosages, lowering the possibility of under- or overdosing. For instance, the CLAD system employs a fuzzy logic controller to deliver propofol and remifentanil while upholding the target degree of anaesthesia (Table 2) [11].

**Table 2:** Pharmacological models using AI TCI: target controlled infusion, CLAD: closed loop anaesthesia delivery system, SVV: stroke volume variation, MAP: mean arterial pressure, NIBP: non-invasive blood pressure, SV: stroke volume

System	Advantages
Diprifusor [8,9]	TCI pumps based on pharmacological model
CLAD [11]	Drug delivery systems to deliver drugs targeting an anaesthetic depth
McSleepy [28,29]	It is an autonomous system that simultaneously balances hypnosis, analgesia, and neuromuscular block
SEDASYS [30]	AI model designed to administer propofol to achieve mild to moderate sedation
Goal-directed fluid therapy models [31-33]	AI algorithm delivering fluid bolus based on urine output, blood pressure, SVV, MAP, etc.
Models for vasopressor titration [34,35]	AI algorithm delivering vasopressors like phenylephrine and norepinephrine based on NIBP, SV, and SVV

Further development in the CLAD system led to pharmacological models that controlled each component of anaesthesia. McSleepy system is capable of administering hypnosis, analgesia, and neuromuscular block simultaneously via administering propofol, remifentanil, and rocuronium. The drug delivery is based on clinical parameters like bispectral index, analog score, and train of four [28,29]. SEDASYS is a computerized sedation system that delivers propofol for mild to moderate sedation by non-anaesthetic physicians (Table 2) [30].

CLAD has been developed for goal-directed fluid therapy based on parameters like mean arterial pressure, urine output, pulse pressure variation, stroke volume variation, or a combination of these [31-33]. Models have been designed to control blood pressure by titration of vasopressors like phenylephrine and norepinephrine using CLAD [34,35].

Mechanical Robots

AI has developed robotic systems to carry out mechanical tasks like intubation, ventilation, and nerve blocks (Table 3). These systems are mostly used in mannequin studies at present but have extreme potential in the future. Da Vinci is the first anaesthesia robot used for fibreoptic endotracheal intubation [36]. The Kepler Intubation System is another robotic system for intubation using a video laryngoscope. It is controlled through a joystick, which is linked to a robotic arm that has a standard video laryngoscope attached to it [37]. Robotic endoscope-automated via laryngeal imaging for tracheal intubation device offers visualization and automated tip placement toward the glottis [38].

**Table 3:** Mechanical robots in anaesthesia psi: pound per square inch, USG: ultrasonography, REALITI: robotic endoscope-automated via laryngeal imaging for tracheal intubation device, SAFIRA: safer injection for regional anaesthesia

Mechanical robots	Application and uses
Da Vinci surgical system [36]	Used for fiberoptic intubations
Kepler intubation system [37]	Used for intubation. It has a joystick-controlled, robotic arm-guided intubation
REALITI [38]	Endotracheal intubation using real-time visualization and robotic placement of endoscope toward the glottis
SAFIRA [41]	Syringes designed to aspirate and stop flow when injection pressure exceeds 15-20 psi
Magellan system [40]	Nerve blocks using USG-guided robotic needle placement
Airway management robot for non-invasive positive pressure mask ventilation [39]	Robot designed for airway management during anaesthesia. It has two arms and a system to fasten the mask. It is a joystick-controlled equipment

A novel anaesthesia airway management robot was recently developed for non-invasive positive pressure ventilation for air management during general anaesthesia. It has two arms, one for lifting the patient's jaw and another one has a mask-fastening system to properly place the mask on the patient [39]. Future studies are needed to evaluate their clinical use.

Some robots have been developed to aid regional anaesthesia and offer better dexterity and precision. Magellan, a robotic arm controlled by a joystick for needle insertion, was used in training in USG-guided regional anaesthesia [40]. Safer injection for regional anaesthesia has the ability to aspirate and stop the flow when injection pressure exceeds the set value [41]. Further development in these systems with AI-enabled programs can ensure patient safety (Table 3).

Finally, AI-automated anaesthetic record-keeping helps to track patient progress, enables communication among healthcare professionals, and offers data for research and quality improvement [4,26]. Manual record-keeping is time-consuming and prone to mistakes, though. AI devices can automatically record vital signs, medication dosages, and other crucial data, enabling the anaesthesiologist to concentrate on patient care. For instance, AIMS keeps a thorough record of the anaesthetic management by collecting and analyzing patient data as well as producing detailed reports [23,24]. By automating record-keeping, it is possible to increase documentation's effectiveness and accuracy, which will raise the standard of care [4].

Limitations and ethical considerations

The use of AI in anaesthesia has enormous potential, but there are certain drawbacks that need to be addressed. AI systems require high-quality data to work effectively, especially those built on machine learning and deep learning concepts. The AI algorithms are trained based on these data, which enables them to develop and enhance their performance over time. However, getting this information can be a challenging task. These data are collected from various sources like anaesthesia machines, patient monitors, and electronic health records. Thus, it is quite difficult to guarantee the accuracy, thoroughness, and consistency of this data. Patient safety may be jeopardized by incorrect AI algorithms caused by incomplete or faulty data [42-44]. Furthermore, because patient information and privacy must be safeguarded, the requirement for enormous amounts of data raises concerns regarding privacy and data security [45,46].

The application of AI in anaesthesia is significantly hampered by technical constraints. Despite their increased sophistication, AI systems still have several limits. For instance, AI systems could have trouble comprehending difficult medical ideas or adjusting to novel circumstances. They are also reliant on the caliber of the data used for their training and programming. AI systems may produce inaccurate predictions or recommendations if they have been improperly developed or if they have been trained on biased or unrepresentative data [42]. Additionally, AI systems lack empathy and human judgment, both of which are essential in the healthcare industry. For instance, depending on the patient's data, an AI system might suggest a certain anaesthetic strategy, but it cannot take into consideration the patient's concerns or anxiety regarding the treatment.

Another significant obstacle to the application of AI in anaesthesia is legal and ethical issues. Several legal and ethical issues are brought up by the usage of AI in healthcare. For instance, who is in charge if a mistake is made by an AI system? Who created the system, the anaesthesiologist who used it, the programmer who created it, or the hospital that put it into use? The law is still ambiguous when it comes to this matter of liability. Patient consent is another ethical issue. Patients must be made aware of the pros and cons of AI and given alternative options if they are not willing. Patients' comprehension of the intricate operations of AI systems can be difficult to convey. Concerns exist over the possibility that AI will take over human professions in the healthcare industry. Even if AI can automate some tasks, the human touch is still essential in healthcare; therefore, its purpose should be to support healthcare personnel rather than to take their place [47].

Despite these difficulties, there are considerable potential advantages of AI in anaesthesia, and actions are being taken to resolve these problems. For instance, research is being done to enhance the quality and security of health data, and guidelines are being produced for the moral application of AI in healthcare [45,46]. AI technological advancements and improved AI system training are addressing technical restrictions. Although there may be many obstacles on the way to the mainstream adoption of AI in anaesthesia, the final destination promises to be transformative, providing better patient care, more efficiency, and new opportunities for research and innovation.

Emerging trends in AI-enhanced anaesthesia

The future of AI in anaesthesia is bright and is anticipated to have notable developments and new application fields. One of the most important future directions is the continued development of AI and machine learning technology. It is anticipated that as these technologies advance, they will become more precise, dependable, and able to handle increasingly challenging tasks. For instance, the rapidly developing subject of deep learning, a subset of machine learning that makes use of neural networks with numerous layers (thus, the "deep" in deep learning), may have important ramifications for anaesthesia. Large volumes of data can be processed using deep learning algorithms, which can then find links and patterns that are too complicated for conventional algorithms to handle. This might improve the ability of AI systems used in anaesthesia to anticipate outcomes and risks of complications by improving their predictive capacities.

Additionally, new potential uses for AI in anaesthesia are being investigated. The management of pain is one such area. It might be difficult to successfully manage pain because it is a complex and subjective feeling. To gauge pain levels and direct pain treatment measures, AI systems may analyze a range of data, including physiological signals and patient-reported results [48]. The therapy of persistent illnesses that need anaesthesia, including chronic pain or palliative care, is another possible area of application [49]. AI has the potential to analyze long-term data and offer insights into the efficacy of various anaesthesia procedures, assisting in the direction of therapeutic decisions.

Another fascinating future avenue is the possible impact of AI on anaesthetic education and training. AI might be utilized to develop lifelike simulations for training reasons, giving pupils the chance to hone their abilities in a secure setting [50]. AI could be used, for instance, to mimic various anaesthesia-related patient reactions, giving students the opportunity to experience and react to a range of situations. AI may also give pupils personalized feedback, highlighting their areas of strength and weakness and making specific suggestions for development [51,52]. This might improve the educational process and better prepare students for the challenges of practical work [53].

AI may also be used to assist in ongoing education for anaesthesiologists who are currently in practice [51,52]. AI might pinpoint areas where the anaesthesiologist could improve and offer customized educational tools by examining data from their practice. This might ensure that anaesthesiologists stay current on the most recent developments in the industry and continually advance their practice.

Anaesthesia has several intriguing opportunities for the use of AI. It is anticipated that AI will play a more significant role in anaesthesia as the industry continues to develop and investigate novel uses for this technology. The application of AI in this sector might have a big influence due to the fact that it has the potential to improve medical care, boost productivity, and advance educational and instructional practices. It is essential, however, to keep in mind that AI should be seen as a tool to aid anaesthesiologists rather than a replacement for them. In the field of medicine, both the human element and specialist knowledge will remain indispensable. AI has the potential to bolster the expertise of anaesthesiologists by offering support and facilitating the making of better-informed judgments.

Future research and development recommendations

Several recommendations for additional training and development can be made in light of the budding importance of AI and machine learning in anaesthesia, but at the same time, hitches and ethical issues need to be addressed. First, further training is essential to improve the sanctuary and eminence of health data. When such instruments are purposefully exploited or abused, regulators have a serious practical problem in that they are powerless to hold offenders accountable through the legal system. This would necessitate the instilling of strong data security procedures as well as the formation of advanced techniques for data collection and scrutiny and legal laws.

Second, the accuracy and comprehension of AI systems should be concentrated on additional research and development. This may require evolving more multifaceted AI algorithms and improving their legibility and precision.

Third, further exploration and research are required to inspect nascent and possible uses for AI in anaesthesia. This might necessitate running randomized trials to evaluate AI's performance and effectiveness in treating chronic illnesses, mitigating pain, and other areas.

Finally, further research must be focused on the possible effects of AI on anaesthetic teaching and training. This could involve creating simulation programs powered by AI and custom feedback systems (as traditionally used in basic and advanced life support training mannequins), as well as researching how efficiently they improve learning results. AI in anesthetic education and training has the ability to provide trainee anesthesiologists with realistic simulations, individualized learning experiences, and data-driven feedback, allowing them to acquire knowledge more swiftly and effectively. The ability of AI to examine large datasets can help with pattern detection and anticipating patient responses, hence improving decision-making skills.

Even though the track to the broad implementation of AI in anaesthesia may be challenging and difficult, the end result promises to be innovative and revolutionary. We can harness the power of AI to completely rejuvenate the field of anaesthesia by concentrating on strengthening data security and quality, increasing the trustworthiness and comprehensive understanding of AI systems, and investigating new application areas.

## Conclusions

AI has the great potential to improve healthcare, enhance productivity, and also help in training and education. It is a transformative technology that can greatly enrich the future of anaesthesia. AI has many applications in anaesthesia, like monitoring vital signs, predicting adverse events, customizing medication doses, and automated record-keeping. The evolution of AI in this field, from the first expert systems to today's cutting-edge AI technologies, demonstrates the power of AI and its potential to totally transform healthcare.

However, the use of AI in anaesthesia has many challenges. Problems with data quantity and quality, technical limitations, and moral and legal issues must all be addressed. AI systems may aggravate bias if the data they are developed on are not accurate. There can be possible breaches of privacy if security protocols are not effectively implemented. High cost can be a limiting factor for its routine use. There is a never-ending debate on the legal responsibility if any error occurs in patient management arising from a fault in AI system. AI systems should be user-friendly, and the interface should be easily understandable. These challenges can be addressed by setting standards for the ethical use of AI in healthcare, improving the reliability and comprehension of AI systems, and ensuring the quality and security of health data.

The future of AI in anaesthesia seems promising. Development in AI and machine learning, new applications, and the potential use in training and education are all exciting future areas. Potential research areas include using AI for chronic illness management and pain management and encouraging anaesthesiologists to better training. If AI is utilized to design authentic lifelike training simulations and offer students with personalized feedback, anaesthesia education might be transformed.
